# Interpretation of custom designed Illumina genotype cluster plots for targeted association studies and next-generation sequence validation

**DOI:** 10.1186/1756-0500-3-39

**Published:** 2010-02-22

**Authors:** Elizabeth A Tindall, Desiree C Petersen, Stina Nikolaysen, Webb Miller, Stephan C Schuster, Vanessa M Hayes

**Affiliations:** 1 Cancer Genetics Group, Children's Cancer Institute Australia for Medical Research, Sydney Children's Hospital, High Street, Randwick, NSW 2031, Australia; 2 Faculty of Medicine, University of New South Wales, Randwick, NSW 2031, Australia; 3 Faculty of Science, University of New South Wales, Randwick, NSW 2031, Australia; 4 Pennsylvania State University, Center for Comparative Genomics and Bioinformatics, University Park, Pennsylvania 16802, USA

## Abstract

**Background:**

High-throughput custom designed genotyping arrays are a valuable resource for biologically focused research studies and increasingly for validation of variation predicted by next-generation sequencing (NGS) technologies. We investigate the Illumina GoldenGate chemistry using custom designed VeraCode and sentrix array matrix (SAM) assays for each of these applications, respectively. We highlight applications for interpretation of Illumina generated genotype cluster plots to maximise data inclusion and reduce genotyping errors.

**Findings:**

We illustrate the dramatic effect of outliers in genotype calling and data interpretation, as well as suggest simple means to avoid genotyping errors. Furthermore we present this platform as a successful method for two-cluster rare or non-autosomal variant calling. The success of high-throughput technologies to accurately call rare variants will become an essential feature for future association studies. Finally, we highlight additional advantages of the Illumina GoldenGate chemistry in generating unusually segregated cluster plots that identify potential NGS generated sequencing error resulting from minimal coverage.

**Conclusions:**

We demonstrate the importance of visually inspecting genotype cluster plots generated by the Illumina software and issue warnings regarding commonly accepted quality control parameters. In addition to suggesting applications to minimise data exclusion, we propose that the Illumina cluster plots may be helpful in identifying potential in-put sequence errors, particularly important for studies to validate NGS generated variation.

## Background

Commercially available genome-wide single nucleotide polymorphism (SNP) arrays and high-throughput "custom designed" genotyping of targeted variants are desirable for biologically focused research. The generation of next-generation sequencing (NGS) *de novo *and resequencing data is contributing to the increased desire for custom, high-throughput arrays for variant validation and determination of allele frequencies [1,2]. For custom designed genotyping, assay reliability and productivity is considered more crucial than perhaps for genome-wide association studies (GWAS). Variants are chosen to answer a specific question, and therefore variant selection is more thoughtful and less redundant.

The Illumina platform (Illumina Inc., San Diego, CA, USA) has proven reliable and efficient for a number of high-throughput genotyping applications using DNA extracted from several sources, [3-9]. VeraCode and BeadArray technologies are used with the GoldenGate assay (Illumina) developed for simultaneous determination of between 96 and 384 (VeraCode) and 96 and 1,536 (BeadArray) variants. GoldenGate chemistry employs the use of allele specific oligo (ASO) hybridisation coupled with fluorescent labelled universal amplification primers for genotype differentiation. In addition to genotype information, automated data analysis provides measures of SNP and sample quality control (QC). Previous studies have adopted guidelines suggested by Illumina for determining assay and sample reliability based on a quality score (GenCall score) calculated by the degree of separation between homozygote and heterozygote clusters [7,10,11]. A value between 0 and 1 is assigned to each call with a score of ≥0.3 and ≥0.25 generally used as the cut-off for the overall SNP and each individual genotype, respectively. In addition to this automated QC, investigators have applied statistical methods such as Hardy-Weinberg equilibrium (HWE) and tests for Mendelian consistency to filter reliable assays [8,10,12-14].

In this study, we assess genotype information generated on custom-designed Illumina 96-SNP VeraCode and BeadArray Sentrix Array Matrix (SAM) assays, for optimal generation of accurate genotyping and NGS generated variant validation. Application of strict analysis parameters may lead to valuable genetic information being lost. Alternatively, limited QC may result in the addition of incorrect genotype calls confounding data analysis. The SNP assays described in this study have the potential of being disregarded or falsely included during analysis based on the current QC selection criteria. We discuss the importance of visually inspecting each cluster plot, particularly for custom designed genotype arrays, and suggest strategies for interpreting data in plots that would normally be discarded during the QC process.

## Methods

### Sample and Genotyping Data Sets

Two sample data sets were used to assess and interpret Illumina-generated genotype cluster plots. The first was part of a gene/pathway targeted human prostate cancer association study. In brief, DNA was extracted from dried blood spots (Guthrie card) and genotyped using a custom 96-plex VeraCode SNP array for 768 samples (unpublished data). In the second study, DNA was extracted from ear clippings of Tasmanian devils (*Sarcophilus harrisii*). DNA was genotyped as part of a validation of sequence variation determined from minimal coverage (0.3× versus 0.5×) of NGS *de novo *sequencing data of two animals using the Roche/454 Titanium chemistry (unpublished data). Genotype analysis was performed using a custom 96-plex SAM array for 96 samples. For both studies, the GoldenGate genotyping procedure was performed as outlined by Illumina [15].

### Data analysis and genotype confirmation

Genotype calls were generated automatically using the GenCall software version 3.1.3. Due to the potential of intra-plate inconsistencies (e.g. variation in fluorescent intensities), the eight VeraCode runs were assessed individually. The genotype cluster plots generated by each VeraCode and SAM assays were visually inspected for quality of calls. Plots that appeared to be "unusually" clustered (ie not following the typical "spread" predicted in regards to software generated HWE or distance between clusters (θ)) were further investigated by selecting samples to assess by direct Sanger sequencing for genotype confirmation. Samples were sequenced using Big Dye Terminator v3.1 (AB, Foster City, CA, USA) chemistry according to manufacturer's guidelines and sequenced on an AB 3730 genetic analyzer (AB).

## Results

### Effect of outliers on cluster formation (VeraCode)

**Figure 1:**
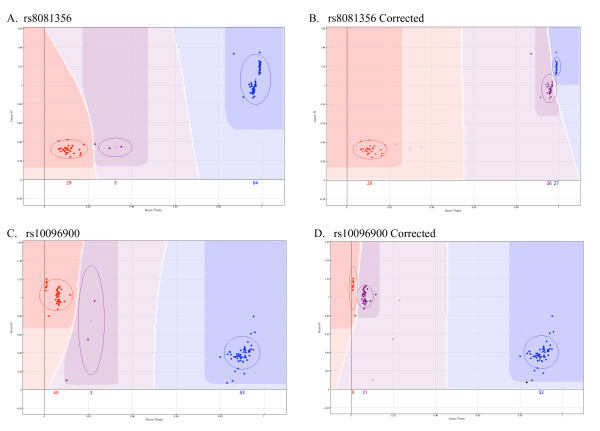
**Effect of outliers on cluster formation**. Exclusion of three outlier samples represented as heterozygous for rs8081356 (A) and rs10096900 (C), corrected genotype clusters for both SNPs (B and D respectively). Correcting the genotype calls reduced the normalised θ (distance between heterozygous and homozygous clusters).

Using the VeraCode technology we observed several consistent examples of SNP cluster plots that appeared to be incorrectly genotyped due to a combination of a short θ and the presence of outlier DNA samples. In the two examples discussed in this study (Figure 1), the presence of just three outlier samples in the original analysis resulted in these outliers being incorrectly genotyped as heterozygous and the true heterozygous samples being grouped with the closely located homozygous cluster (Figure 1A and 1C). Removal of these outliers corrected the genotype calls (Figure 1B and 1D), yet reduced the GenCall score from 0.52 to 0.45 for rs8081356 (Figure 1A and 1B, respectively) and 0.67 to 0.41 for rs10096900 (Figure 1C and 1D, respectively). A measure of HWE for these two SNPs, revealed that the original analysis was not within the expected distributions (P = 1.707778E-20 and 7.727E-21 for rs8081356 and rs10096900, respectively) and thus may have been excluded on this basis. Exclusion of the outlier samples corrected for the deviation from HWE.

### Two-cluster autosomal and non-autosomal SNP calling (VeraCode)

**Figure 2:**
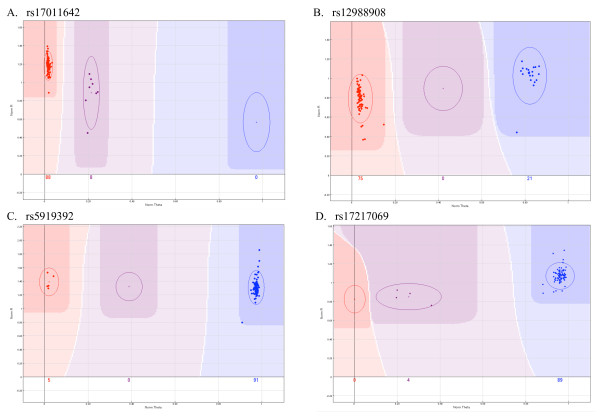
**Two cluster calling**. Rare variant and two-cluster genotyping was successful for the majority of samples assessed on the VeraCode platform as demonstrated in rs17011642 (A). Incorrect rare variant calling was observed for SNP rs12988908, with the heterozygous samples being called homozygous for the mutant allele (B). Non-autosomal variants were correctly genotyped in most cases as demonstrated in SNP rs5919392 (C). Only one X-linked SNP, rs17217069 was incorrectly genotyped with the mutant allele being called heterozygous.

For rare and single copy variants (male Y- and X-chromosomes), genotyping technologies must accurately perform two-cluster calling. In this study successful two-cluster calling was observed for both rare autosomal (i.e. homozygous wild-type and heterozygous; rs17011642 Figure 2A) and single copy X-linked variants (rs5919392 Figure 2C) as well as incorrect two-cluster calling for both scenarios (rs12988908 Figure 2B and rs17217069 Figure 2D). The assay was termed "incorrectly genotyped" when one or more cluster plots across the eight VeraCode plates for that variant was miss-called. Miss-calling was usually represented by the heterozygous samples being assigned the homozygous mutant genotype for rare variants (Figure 2B) and *vice versa *for the X-linked variants (Figure 2D). Although rs12988908 would have been rejected during QC based on deviation from HWE (P = 1.529597E-23), but not GenCall score (0.72), rs17217069 on the X chromosome, would not have been detected as false based on this selection criteria (HWE P = 0.82, GenCall = 0.67). Incorrect two-cluster calling was observed in just 4/17 (24%) of rare autosomal variants and 1/9 (11%) of X-linked variants.

### Undefined cluster distribution in a verified sequence (VeraCode)

**Figure 3:**
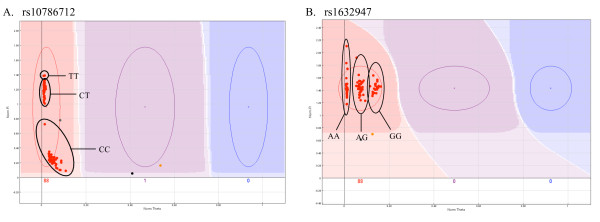
**Undefined cluster distribution for verified human SNPs (VeraCode)**. The GenCall software failed to define genotypes for two verified human SNPs, rs10786712 (A) and rs1632947 (B) genotyped on the VeraCode platform. Samples were poorly separated into three groups along the Y- and X-axis of the cluster plot. These three groups represented the three different genotypes as indicated.

In the two examples highlighted in this section of the study, rs10786712 and rs1632947, all samples were genotyped by the automated GenCall software as homozygous, despite having a validated minor allele frequency >0.39 [16]. For both SNPs, the software generated three marginally separated groups within the one genotype, spread along the Y- and X-axis of the rs10786712 and rs1632947 cluster plots respectively (Figures 3A and 3B). Direct Sanger sequencing confirmed the presence of the three different genotypes as indicated in Figure 3A and 3B. Sequencing did not reveal any other obvious variation in the sequence surrounding these SNPs that might explain the unusual clustering.

### Undefined cluster distribution in a de novo NGS generated SNP array (SAM)

**Figure 4:**
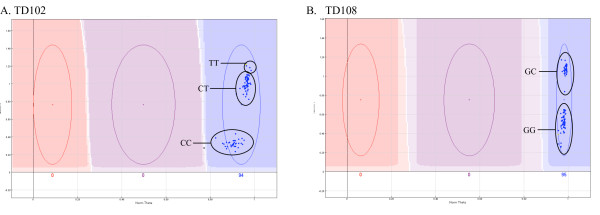
**Undefined cluster distribution in a *de novo *NGS generated SNP array (SAM)**. Failure to define separate genotype clusters for newly identified variants TD102 (A) and TD108 (B) was due to incorrect sequence input into the GoldeGate assay design software. The failure of one ASO to bind resulted in the different genotype groups being separated by a difference in fluorescence intensity of one allele along the Y-axis of the cluster plot.

In a 96-plex SAM array generated to verify variants predicted from limited (0.3× versus 0.5× coverage) *de novo *sequencing, only a single SNP was classified as a genotype failure by the software. We defined 19 plots as failures due to single allele calling. In the majority of such cases (79%, 15/19), direct Sanger sequencing revealed a single nucleotide error in the NGS generated data surrounding the putative SNP. In the examples highlighted in this study (TD102 Figure 4A and TD108 Figure 4B), a single nucleotide immediately adjacent to the putative SNP was mistakenly absent from the NGS generated data, subsequently leading to incorrect ASO design for the custom SAM array. The Illumina cluster plots revealed partial segregation along the Y-axis of the plot, which were confirmed as different genotype groups (Figure 4A and 4B). It should be noted that the remaining 4/19 genotyping failures remain undetermined, as Sanger sequencing confirmed the NGS-generated sequence.

## Discussion

Large-scale custom-designed genotyping studies have been made feasible by the development of high-throughput technologies and improvements of automated genotype calling software. In this study we demonstrate how simple applications for interpreting automatically generated Illumina cluster plots can be used to avoid spurious genetic associations as well as optimising data inclusion and interpretation, particularly for custom designed target arrays.

Variation in quality and quantity of DNA input, is largely unavoidable in large-scale studies. In order to limit the effect potential outlier DNA samples may have on genotype calls, it is important to visually inspect the genotype plots and exclude outliers as demonstrated in this study (Figure 1). In addition to illustrating the importance of visual analysis, these examples (rs8081356 and rs10096900) emphasise the need to adopt QC criteria that extends beyond the GenCall score calculated by the Illumina analysis software. For both variants, the GenCall score decreased when the correct genotypes were applied (Figure 1B and 1D), and is therefore not an optimal measure of assay reliability in such scenarios.

Investigating the disease-causing potential of rare variants (that are often missed on larger scale GWAS [17]), can prove problematic for genotyping arrays. In this study we found the Illumina VeraCode and SAM technologies to be highly reliable for rare variant and single allele (male sex chromosome) two-cluster calling. However, we also highlight in this study, potential QC problems that may arise for two-cluster genotyping. We observed that HWE was successful as a measure of assay reliability when interpreting autosomal rare variant miss-calling, but was not suitable for single allele X-linked variants. For this application, visual inspection of cluster plots in combination with prior knowledge of the variant statistics (e.g. location and population-specific allele frequency) would provide an adequate means of determining assay success.

A growing number of laboratories are employing the use of NGS technology to sequence as yet unclassified genomes, as well as resequencing of human and human disease-associated genomes. From the predicted sequence variants generated by these projects, custom-designed arrays are being employed for validation and frequency determination. Although the optimal coverage required to distinguish true sequence variation is highly debatable, and dependent on the type of NGS platform used, high-throughput genotyping platforms allow for rapid, cost-effective validation at even minimal coverage. The SAM array discussed in this study was developed from long-read NGS information generated from minimal coverage of two samples of an as yet unsequenced species (unpublished data). In both the VeraCode assay, custom designed to genotype 96 confirmed human variants, and the SAM array, designed to verify newly identified variants from *de novo *generated NGS data, we observed several examples of individual clusters being partially segregated within the one genotype group (Figure 3 and 4). Although this cannot be attributed to miss-calling on the VeraCode assays (Figure 3), the unusually grouped variants described on the SAM array (Figure 4) were due to incorrect oligo design as a result of miss-interpretation of *de novo *generated NGS data. For variants TD102 and TD108, NGS results suggested the presence of an additional nucleotide immediately adjacent to the variant site, which was the same as the alternative allele of the putative variant. Direct Sanger sequencing confirmed that this additional nucleotide was not present and hence we observed varying binding capabilities of only one ASO, resulting in the fluorescent intensity of samples containing a mutant allele being greater than those without. In addition to confirming the presence of a variant at these locations, the unusually clustered assays on the SAM array, prompted closer inspection (visual and Sanger sequencing) which lead to the identification of NGS-generated sequencing error. We therefore suggest that visual inspection and closer investigation of unusually clustered scatter plots, may provide information that exceeds the initial goal of SNP validation and is a complimentary tool for NGS data validation.

## Conclusion

We demonstrate in this study, applications to optimise and improve the efficiency of data analysis generated using the Illumina GoldenGate chemistry using logical interpretation of both rare and common genotyping data. We also present this platform as a successful tool for NGS variant validation, which is applicable to even limited sequence coverage.

## Competing interests

The authors declare that they have no competing interests.

## Authors' contributions

WM and SCS provided analysis of NGS data, EAT, DCP and SN performed genotyping and Sanger sequencing analysis; EAT, DCP, SN and VMH were involved in data analysis; and EAT and VMH participated in study design and manuscript drafting. All authors read and approved the final manuscript.
